# Supplementary material for: Pollution exacerbates China’s water scarcity and its regional inequality
Source: Nat Commun. 2020 Jan 31;11:650. doi: 10.1038/s41467-020-14532-5 (PMC6994511; doi:10.1038/s41467-020-14532-5)
Supplement: Supplementary file 2 — Supplementary Information [file 41467_2020_14532_MOESM2_ESM.pdf]

Supplementary Information

For

Pollution exacerbates China's water scarcity and its regional inequality

Ma et al.

Supplementary Notes

Supplementary Note 1: Carrying capacity of water resources taking quality into account

The impact of water quality on domestic and production water uses can also be analyzed using the concept of water carrying capacity, which refers to the maximum population or economic scale that can sustainably be supported by the available water resources[1,2]. Methods for the carrying capacity evaluation of water resources are diverse, and a comprehensive review can be found in a recent study[2]. In our research, we evaluated the water carrying capacity in terms of a multiplier of present-day population or production scale that can be sustained by the water endowment and quality conditions. The quantity-based water carrying capacity for sector *i*, denoted as $C\mathrm{qua}_i$, is defined as follows:

$$C\mathrm{qua}_i = \frac{Q - EFR}{D_i} \quad \text{(S1)}$$

where Q is water availability, EFR is the environmental flow requirement, which is defined 80% of water availability as in water scarcity assessment, D_i is water withdrawal for sector *i*. $C\mathrm{qua}_i$ represents the maximum population (i = 3) or economic scale (i = 1 or 2) in terms of a multiplier of the current scale when other sectoral water uses are not considered. The carrying capacity, which considers all sectoral water uses, denoted as $C\mathrm{qua}$, is defined as a multiplier of the current scale of both population and economic development:

$$C\mathrm{qua} = \frac{Q - EFR}{\sum_i D_i} \quad \text{(S2)}$$

When the quality aspect is taken into account, the dilution approach is used, as in the quality-based water scarcity assessment in the main body of the article; the dilution approach translates inadequate water quality into an additional water quantity requirement to obtain the adequate quality for the various water uses. Similarly, the water carrying capacity for sector *i*, which considers the combined effects of water quantity and quality, denoted as $C\mathrm{com}_i$, is defined as follows:

$$C\mathrm{com}_i = \frac{Q - EFR}{D_i + dq_i} \quad \text{(S3)}$$

where dq_i is the extra water required for dilution to obtain acceptable quality for water use sector *i* (see Equation 3 in the main body of the article). The carrying capacity, which takes into account all sectoral water uses with the combined effects of water quantity and quality, denoted as $C\mathrm{com}$, is defined as follows:

$$C\mathrm{com} = \frac{Q - EFR}{\sum_i D_i + \sum_i dq_i} \quad \text{(S4)}$$

A water carrying capacity *C*qua or *C*com larger than 1 would mean that the water resources are able to sustainably support the current population and economic scale; this would correspond to low water scarcity, with a WSqua or WScom of less than 1. In contrast, a water carrying capacity *C*qua or *C*com below 1 would indicate the insufficient carrying capacity of water resources to support the present-day population and economic scale; this would correspond to a moderate-to-severe water scarcity issue, with a WSqua or WScom of over 1. Thus understood, the value of *C*qua or *C*com is the reciprocal of the value of WSqua or WScom.

As an example, Supplementary Fig. 12 shows the water carrying capacity in terms of a multiplier of present-day population and economic scale at the first-order basin level on an annual basis. In this case, as the agricultural water use constitutes a significant portion of the total water use, the carrying capacity for agriculture is the lowest in all the sectors. The water resources in the six North China basins are incapable of sustaining the agricultural scale alone with both *C*qua and *C*com below 1. In the most-water-scarce Haihe River basin, the water carrying capacity is not

able to support each of the sectoral water uses alone. The carrying capacity *C*com in all the six North China basins and the Southeast River basin is less than 1, indicating an insufficient capacity of water resources for sustaining the population and economic activities in these basins (Supplementary Fig. 12h). While the quantity is sufficient for supporting human's water needs and environmental flow requirement in the Southeast River basin, its inadequate water quality makes the quality included carrying capacity below 1. This is consistent with the water scarcity condition of these basins, as shown in Fig. 2c. Supplementary Fig. 12 also shows that inadequate water quality reduces the carrying capacity of water resources.

Supplementary Note 2: Water scarcity assessment considering seasonal variability for the environmental flow requirement

An environmental flow requirement (EFR) will show seasonal variability. The role of seasonal variability for an EFR in a water scarcity assessment has been highlighted in recent studies[3,4]. In the main text, we presented seasonal and monthly water scarcity levels with a fixed proportion of EFR for consistency with annual analyses. Here, the impact of seasonal variability for EFR is analyzed, assuming the changing proportions of water flows for monthly EFRs using a variable monthly flow method[4]. This variable monthly flow method has been shown to be a valid and straightforward method for implementation in global models[4]. The variable monthly EFR was defined for low-flow, intermediate-flow, and high-flow conditions. In the low-flow months, when the mean monthly flow (MMF) was below 0.4 times the mean annual flow (MAF), the EFR was considered to be 0.6 times the MMF. In the intermediate-flow months, when the MMF was between 0.4 and 0.8 times the MAF, the EFR was considered to be 0.45 times the MMF. In the high-flow months, when the MMF was over 0.8 times the MAF, the EFR was considered to be 0.3 times the MMF. The corresponding WSqua and WScom were calculated using Equations 1-4 in the main body of the article, but with monthly variable EFR. The monthly water scarcity that takes into account the seasonal variability of EFR was classified into two levels based on the value of WSqua or WScom: no water scarcity (< 1.0), and water scarcity (> 1.0). This water scarcity definition is generally less stringent than the one defined as 80% of a water resource allocated to an EFR, as found in the main body of the article, since a larger share of a particular water resource would be considered available for human water uses in this variable EFR case. Supplementary Fig. 13 shows the results of the water scarcity assessment considering variable seasonal EFR on a monthly basis. The inclusion of seasonal variability for an EFR has an impact on the seasonal water scarcity (Supplementary Fig. 13a) because the proportion of EFR directly impacts the availability of water resources for human uses. Nevertheless, regional inequality of seasonal water scarcity seems insensitive to the seasonal variability for EFR in our case (Supplementary Fig. 13b).

Supplementary Note 3: Water quality guidelines for sectoral water uses

Quality influences the water suitability for a specific use. There have been a number of different water quality guidelines related to different water uses, which are not wholly consistent because of the wide variability in field conditions. Our water scarcity assessment, done at a national scale, requires effective but practical water suitability guidelines, particularly considering the limited availability of water quality measurements at the national level. The selection of water quality parameters indicating water suitability for specific uses is based on both data availability and relevance. In this study, the quality requirements for sectoral water uses were based on three standard water quality measures: chemical oxygen demand (COD), ammonium nitrogen (NH_4^+-N), and electrical conductivity (EC). Although these quality requirements are not comprehensive, these three parameters are the main water quality indicators that are widely available at the national level, and generally, can well represent the characteristics of a water supply for its suitability for specific uses. The maximum water quality thresholds for each specific sectoral use, which are based on environmental quality standards for surface water in China and on FAO guidelines, are provided in the main body of this article.

Water quality requirements *Cmax* in Equation 3 are defined for agricultural, industrial, domestic and eco-environmental compensation uses. Though quality requirements may present difference for water uses at a higher resolution in one sector, categorization of sectoral water uses for quality-induced water scarcity assessment in this nationwide study is based on data availability and best practices in China. For instance, drinking and toilet flushing water uses (both in the domestic water use sector) may differ in water quality requirements. However, the extra costs for building and maintaining dual water distribution systems to households and residents' unwillingness to use recycled water are large barriers to extensive use of recycled water for domestic use. Domestic water is mostly supplied to households by water supply networks, without differentiating drinking and other domestic uses. Therefore, in practice, quality requirements for various domestic water uses, including drinking, washing and toilet flushing, do not present any difference due to current water supply systems. Specific industries and irrigated crops may also have different water quality requirements. However, because data for both water uses and quality requirements with sectors of more detailed categories are not readily available, it is difficult to implement quality included water scarcity assessment with more specified water use categories.

Supplementary Note 4: Sectoral water withdrawal data

The annual sectoral water withdrawal data at the province-level in China for the period 2012-2016 were downscaled to generate monthly grid cell data. The downscaling was made by disaggregating the statistical data from the provincial units to grid units based on multi-sourced information.

Agricultural water withdrawal is mainly used for irrigation, and hence, it was disaggregated according to irrigation information. Water withdrawal $da_{j,m}$ in grid cell j in month m was calculated as follows:

$$da_{j,m} = \frac{IR_{j,m} \times AR_j + II_{j,m} \times AI_j}{\sum_{m=1}^{12} \sum_{k=1}^{N} (IR_{k,m} \times AR_k + II_{k,m} \times AI_k)} Da \qquad \text{(S5)}$$

where AR_j and AI_j are, respectively, the areas of rice and non-rice croplands (Supplementary Fig. 14b); $IR_{j,m}$ and $II_{j,m}$ are the net irrigation water requirements in month m for corresponding croplands; Da is the annual agricultural water withdrawal in the province where grid cell j is located; and N is the number of grid cells in that province. AR_j and AI_j were extracted using the 2015 national land use map, with an initial spatial resolution of 100 m, and interpreted from Landsat TM and ETM+ images[5,6]. Following Smith's approach in the model CROPWAT[7,8], the net irrigation requirement $I_{j,m}$ (for both $IR_{j,m}$ and $II_{j,m}$) was estimated as the difference between the reference crop evapotranspiration $EP_{j,m}$ and the effective precipitation $PE_{j,m}$:

$$I_{j,m} = k_c \times EP_{j,m} - PE_{j,m} \qquad \text{(S6)}$$

where k_c is the crop coefficient, and $EP_{j,m}$ is the monthly reference evapotranspiration (ET_o), calculated as the sum of daily ET_o, which was estimated using the FAO Penman-Monteith equation[9]. $PE_{j,m}$ was computed through a segmented function of precipitation, as demonstrated by Döll and Siebert[8].

In this research, three predominant Chinese crops—rice, maize, and wheat—were chosen to determine the grid cell-level crop coefficient k_c in Equation S6. Single- and double-cropped areas were identified by examining the province-level yields of the crops and areas of different croplands. The double-cropped areas were assumed to be restricted to tropical, subtropical, and warm temperate zones in China. The single- and dual-crop coefficients k_c for rice in China were determined according to previous results using the FAO-56 approach by Peng et al.[10]. The growing periods and k_c for non-rice crops (including a single-cropped coefficient for spring maize, and a dual-cropped coefficient for winter wheat and spring maize intercropping) were determined based on previous research on the North China Plain by Gao et al.[11] and Liu and Luo[12].

Annual industrial water withdrawal was downscaled according to maps of the industrial gross domestic product (GDP) at the grid cell-level (Supplementary Fig. 14c). The map of industrial GDP was generated by disaggregating the province-level data through a proportional sharing method based on nighttime brightness-weighted areas of

industrial lands. Here, we used satellite-derived nightlight data because of the well-documented and significant quantitative correlation between socioeconomic variables and anthropogenic nocturnal brightness in areas of high-density human activity[13,14]. The industrial water withdrawals were assumed to be uniformly distributed among all the months of a year. The monthly industrial water withdrawal di_j in grid cells j was estimated as follows:

$$di_j = \frac{GDP_j}{12\sum_{k=1}^{N} GDP_k} Di \tag{S7}$$

where GDP_j represents the industrial GDP in grid cell j, and D_i is the annual industrial water withdrawal in the province where grid cell j is located.

Annual domestic water withdrawal was disaggregated according to spatial urban and rural population distributions and monthly water use factors. Province-level rural and urban populations were downscaled into the grid cell data. The rural population was disaggregated based on the rural residential areas in the grid cells, and the urban population was disaggregated based on the urban areas in the grid cells, as weighted by nighttime light (similar to industrial GDP downscaling) (Supplementary Fig. 14d). A monthly factor for domestic water use for each grid cell $W_{j,m}$ was calculated following Wada et al.[15] and Voisin et al.[16]:

$$W_{j,m} = \left(\frac{T_{j,m} - T_{avg}}{T_{max} - T_{min}} \times R + 1\right)/12 \tag{S8}$$

where $T_{j,m}$ is the average temperature in month m in grid cell j; T_{avg}, T_{max}, and T_{min} are the average, maximum, and minimum monthly average temperatures, respectively; and R is the amplitude factor that quantifies the variation of domestic water use between the warmest and coldest months, which, following the work of Huang et al.[17], was set at 0.2 for this study. Domestic water withdrawal $dd_{j,m}$ in grid cell j for month m was estimated as

$$dd_{j,m} = \frac{W_{j,m} \times Pop_j}{\sum_{m=1}^{12}\sum_{k=1}^{N}(W_{k,m} \times Pop_k)} Dd \tag{S9}$$

where Pop_j is the total population in grid cell j, including both rural and urban residents, and Dd is the annual domestic water withdrawal in the province where grid cell j is located.

According to the China Water Resources Bulletin, eco-environmental compensation water withdrawal is mainly used for irrigating green spaces and replenishing dry rivers and lakes in urbanized areas. This sectoral water withdrawal represents only 2% of China's total water withdrawal. As it is difficult to discern green spaces and water bodies in urbanized areas based on available land use maps, we assumed that water withdrawal for eco-environmental compensation is proportional to the size of urbanized areas in the grid cells (Supplementary Fig. 14e). The eco-environmental compensation water withdrawals were assumed to be uniformly distributed among all months within a year. Monthly eco-environmental water withdrawal de_j in grid cell j can be estimated as follows:

$$de_j = \frac{AU_j}{12\sum_{k=1}^{N} AU_k} De \tag{S10}$$

where AU_j represents the size of the urbanized areas in grid cell j, and De is the annual eco-environmental water withdrawal in the province where grid cell j is located.

China's total annual water withdrawals for the four sectors at the grid cell-level are shown in Supplementary Fig. 14f.

Supplementary Note 5: Data summary for water scarcity assessment

Based on Equations 1-4 in the article, water scarcity assessment requires three main datasets, i.e. water availability, sectoral water use and water quality. All the data sources required for compiling these three datasets are listed in Supplementary Table 1, together with their summarized information.

Supplementary Tables

Supplementary Table 1 Data sources for water scarcity assessment in China

For datasets	Data	Resolutions	Sources and other remarks
Water availability	Statistical available water resources	At the provincial level on an annual basis	Data were referred from China Water Resources Bulletin (2012-2016), which are officially published by Ministry of Water Resources of China. Data are available at http://www.mwr.gov.cn/sj/tjgb/szygb/.
	Water resources availability simulation results by the variable Infiltration Capacity (VIC) hydrologic model	At the 0.25 × 0.25 arc-degree level on a monthly basis	Simulation data were validated with gauge measurements across China and provided by Zhang and Tang, A long-term land surface hydrologic fluxes and states dataset for China. *J. Hydrometeorol*. 15, 2067–2084 (2014).
Sectoral water use	Provincial agricultural, industrial, domestic and eco-environmental water uses	At the provincial level on an annual basis	Data were referred from China Water Resources Bulletin (2012-2016), which are officially published by Ministry of Water Resources of China. Data are available at http://www.mwr.gov.cn/sj/tjgb/szygb/.
	Crop production	At the provincial level on an annual basis	Data were referred from China Rural Statistical Yearbook (2015), which are officially published by Ministry of Water Resources of China. Data are available at China Statistical Yearbooks Database (http://data.cnki.net/).
	Provincial rural and urban population and industrial domestic product data	At the provincial level on an annual basis	Data were referred from China Statistical Yearbook (2012-2016), which are officially published by National Bureau of Statistics of China. Data are available at China Statistical Yearbooks Database (http://data.cnki.net/).
	National land use maps	At the spatial resolution of 1 × 1km on an annual basis	Land use maps (2010, 2015) were produced from Landsat Thematic Mapper (TM) and Enhanced Thematic Mapper Plus (EMT+) images using a supervised classification method in cooperation with ground truth verification. Detailed information can be found in Liu et al., Spatiotemporal characteristics, patterns, and causes of land-use changes in China since the late 1980s. *J. Geogr. Sci.* 24, 195–210 (2014). Data are provided by Institute of Geographic Sciences and Natural Resources Research, Chinese Academy of Sciences and available at http://www.geodata.cn/.
	Nighttime light brightness	At the spatial resolution of 30 arc-seconds on an annual basis	DMSP/OLS-derived nightlight data were provided by the National Oceanic and Atmospheric Administration (NOAA), National Centers for Environmental Information (NCEI). Data were inter-calibrated using the second-order regression model to reduce the yearly variations and differences among

			sensors. Data are available at https://ngdc.noaa.gov/eog/dmsp.html.
	Climate variables	At the site level on a monthly basis	Climatic data (2012-2016) including monthly mean daily minimum temperature, maximum temperature, relative humidity, wind speed and sun hours across 742 sites were provided by China Meteorological Data Network, China Meteorological Administration. Data are available at http://data.cma.cn.
Water quality	Chemical oxygen demand concentration, ammonium nitrogen concentration and electrical conductivity in surface water	At the sampling site level on a monthly basis	Water quality data (2012-2016) were provided by the national environmental monitoring network including 2360 sampling sites. The three water quality indicators were measured according to “Technical Specifications Requirements for Monitoring of Surface Water and Wastewater in China”, issued by Ministry of Ecology and Environment, for guidance. Water quality monitoring data of a selection of sites are publicly available from the website http://www.cnemc.cn/sssj.

Supplementary Figures

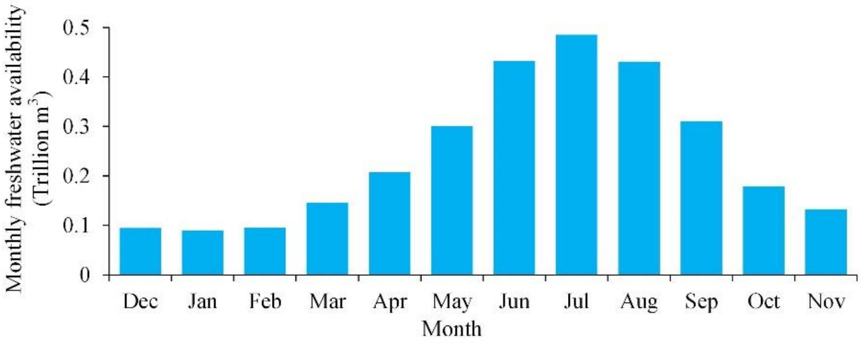

Supplementary Figure 1. The monthly variations in China's freshwater resource availability according to the mean for the five-year period from 2012 to 2016. Source data are provided as a Source Data file.

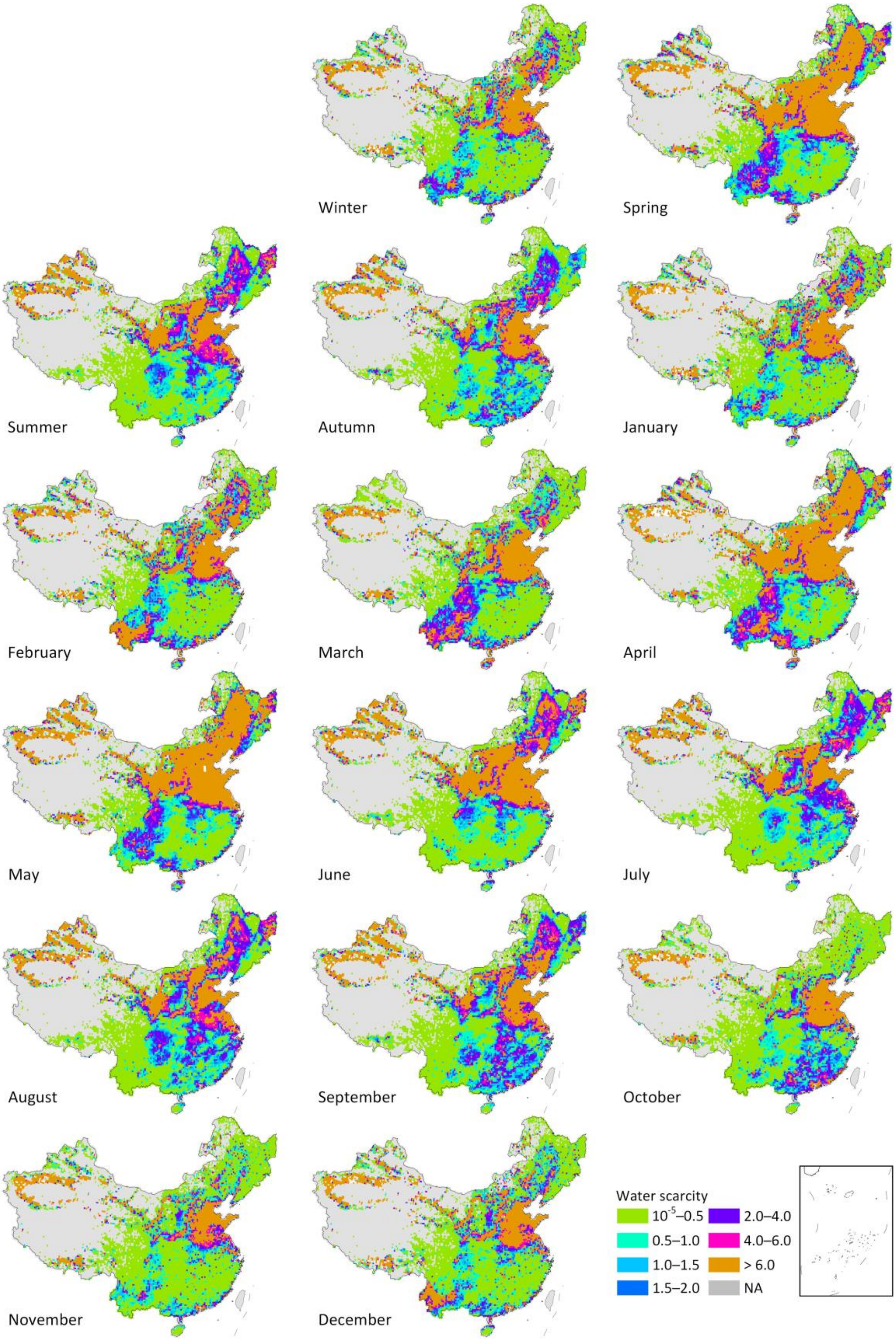

Supplementary Figure 2. Spatial distributions of quality included water scarcity (WScom) in China with a spatial resolution of 0.25 × 0.25 arc-degrees at different time scales. Source data are provided as a Source Data file.

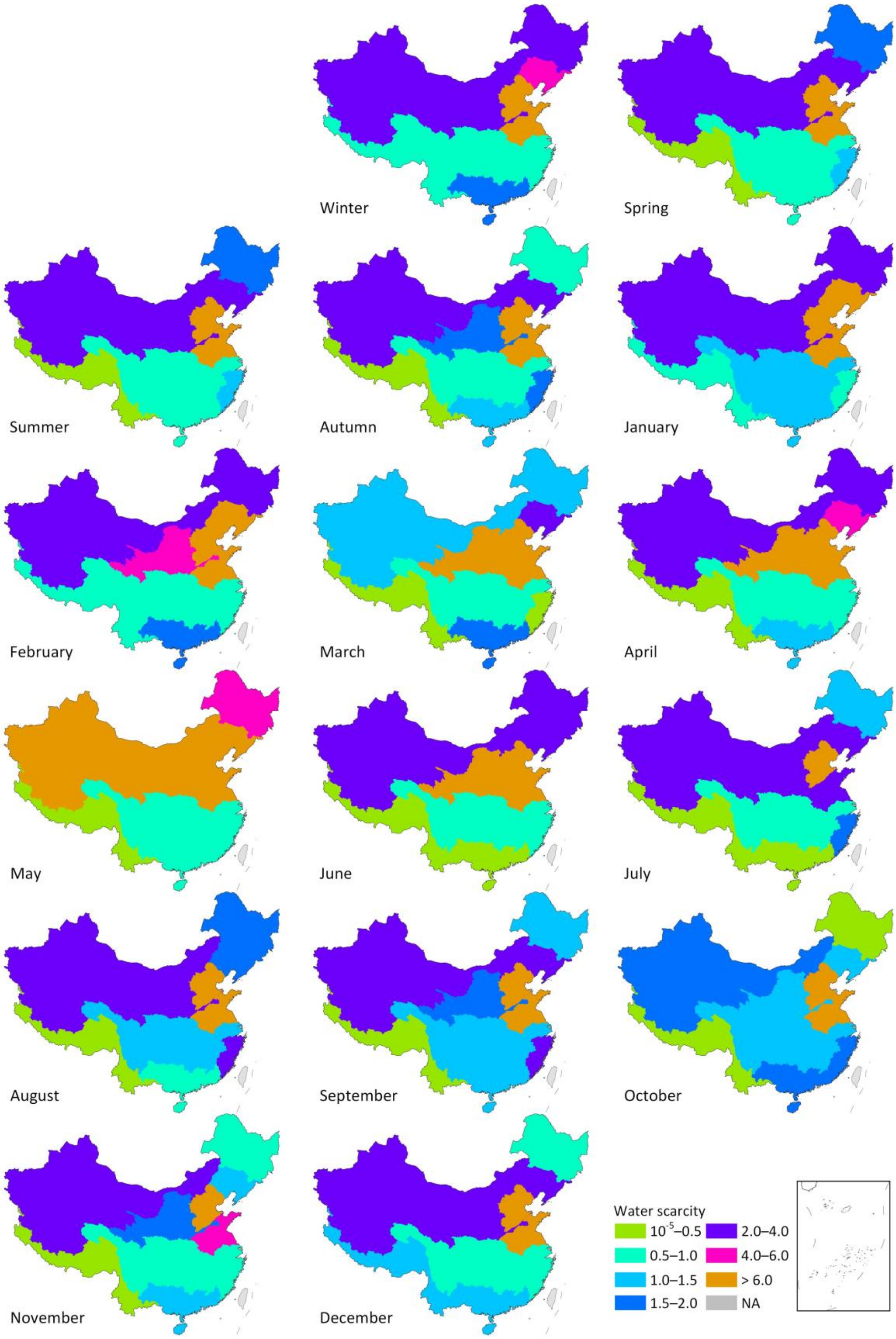

Supplementary Figure 3. Quality included water scarcity (WScom) across first-order basins in China at different time scales. Source data are provided as a Source Data file.

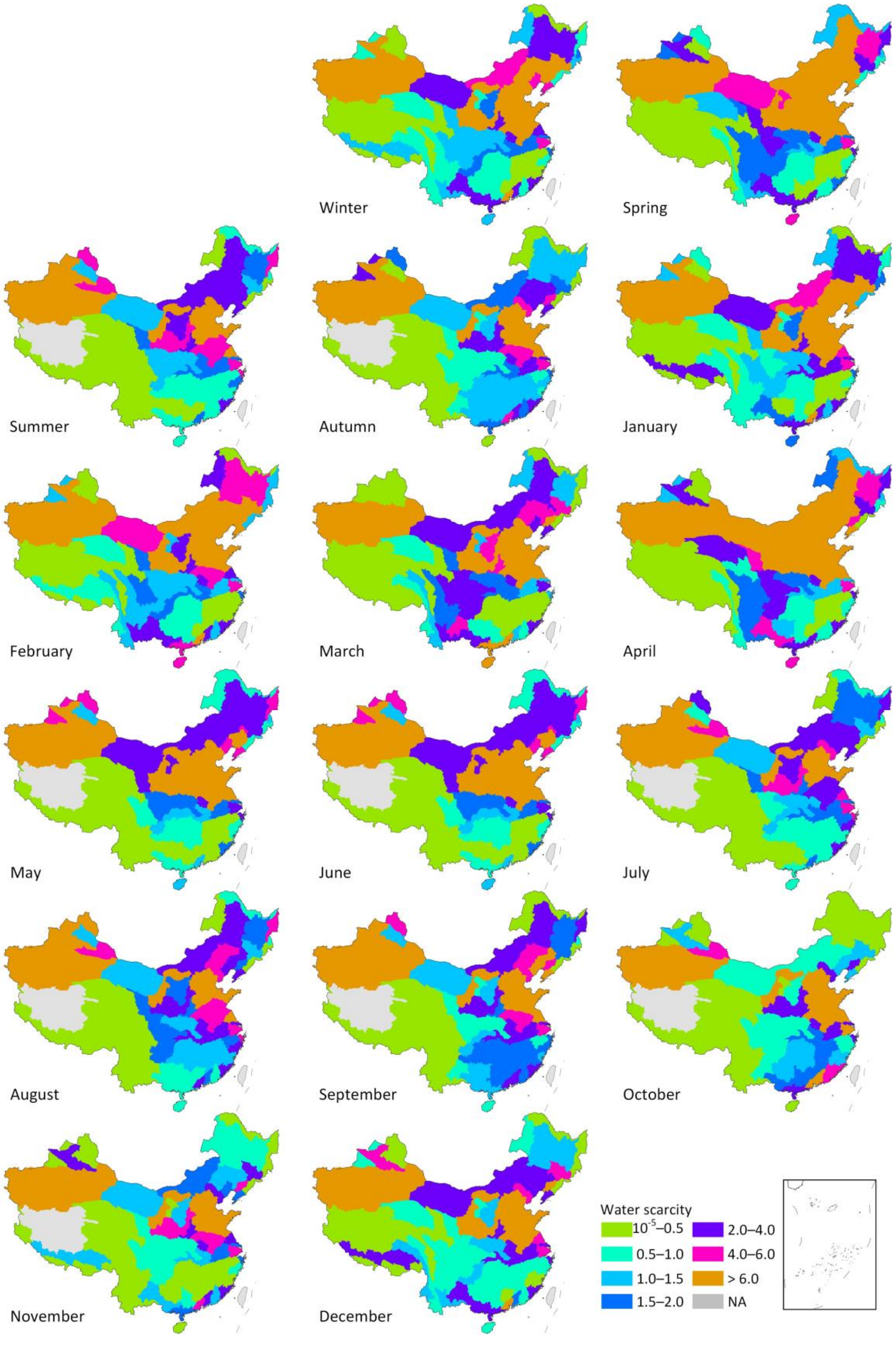

Supplementary Figure 4. Quality included water scarcity (WScom) across second-order basins in China at different time scales. Source data are provided as a Source Data file.

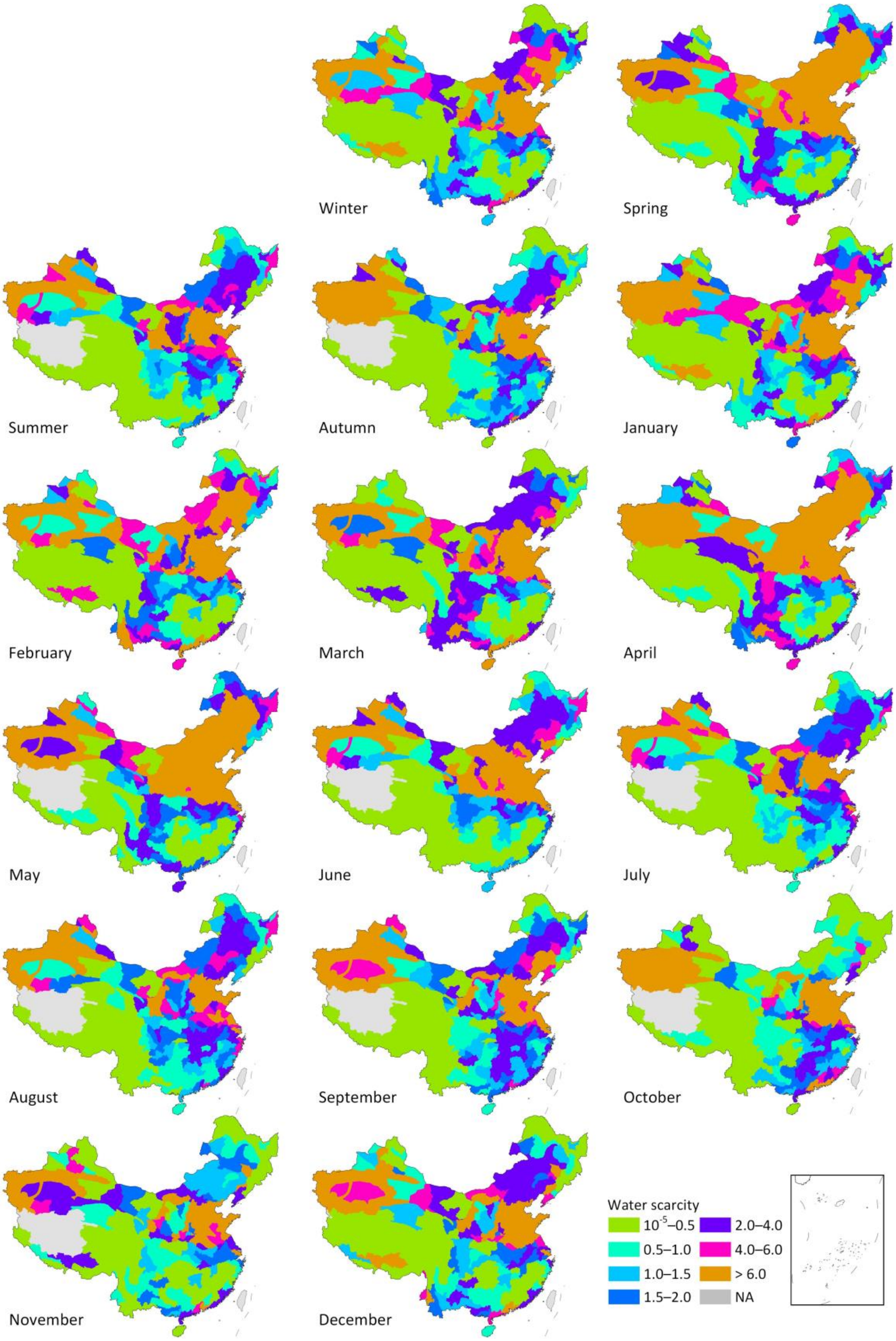

Supplementary Figure 5. Quality included water scarcity (WScom) across third-order basins in China at different time scales. Source data are provided as a Source Data file.

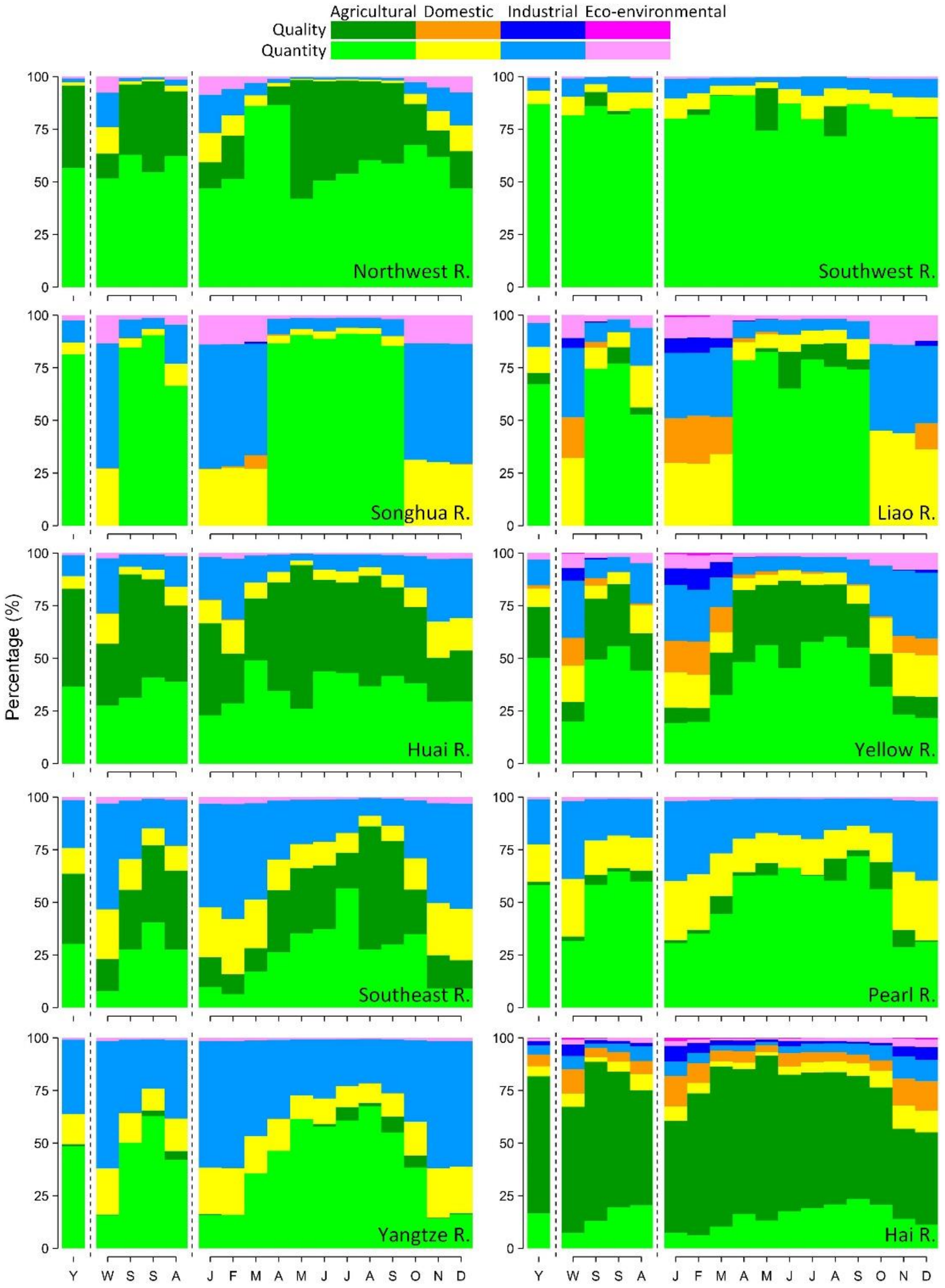

Supplementary Figure 6. Proportions of sectoral water scarcity levels in China across 10 first-order basins. The setup of labels on the x-axis is the same as in Fig. 3. Source data are provided as a Source Data file.

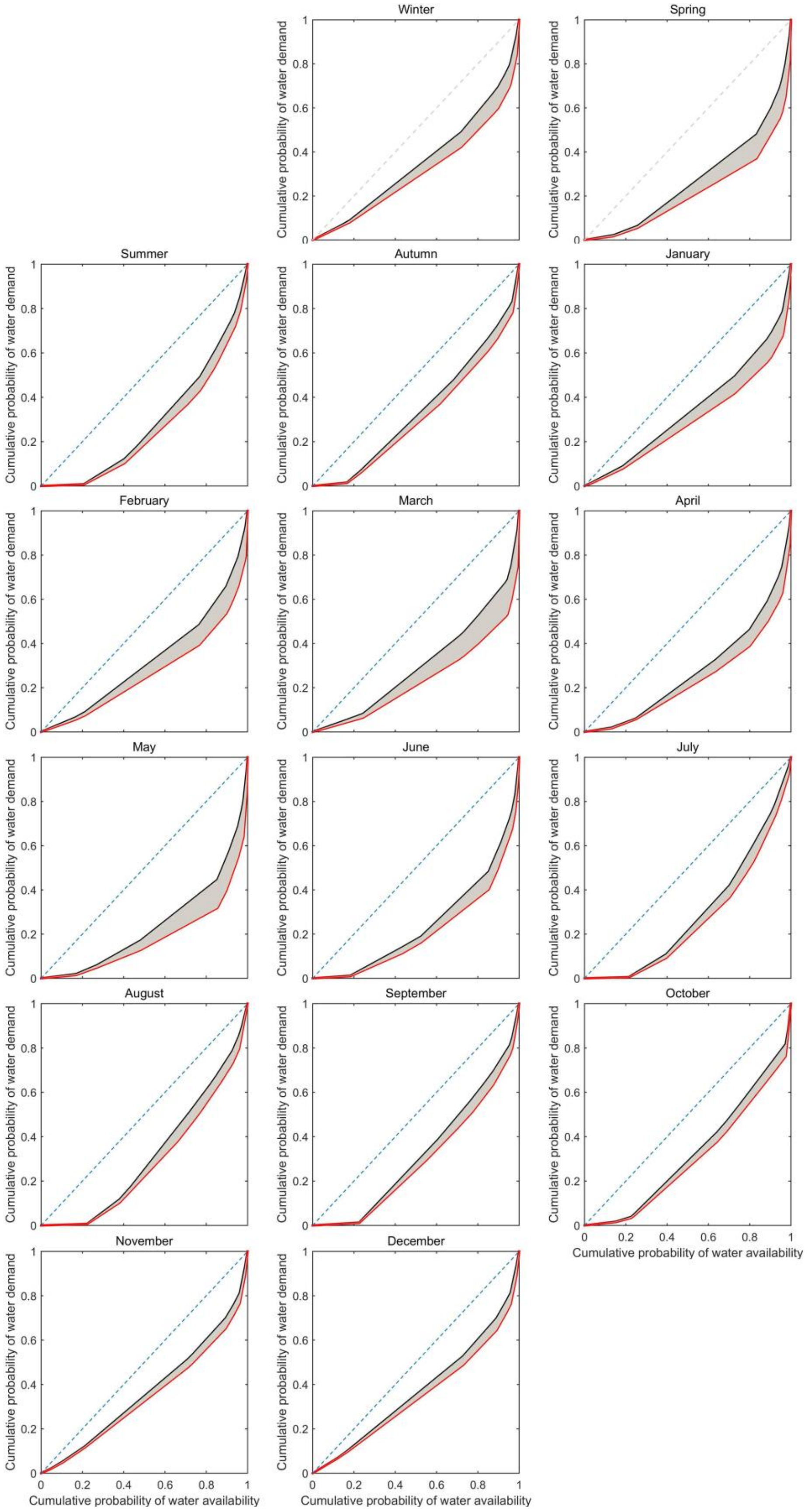

Supplementary Figure 7. Cumulative probability of water availability against cumulative portability of water withdrawals at the level of the first-order basin.

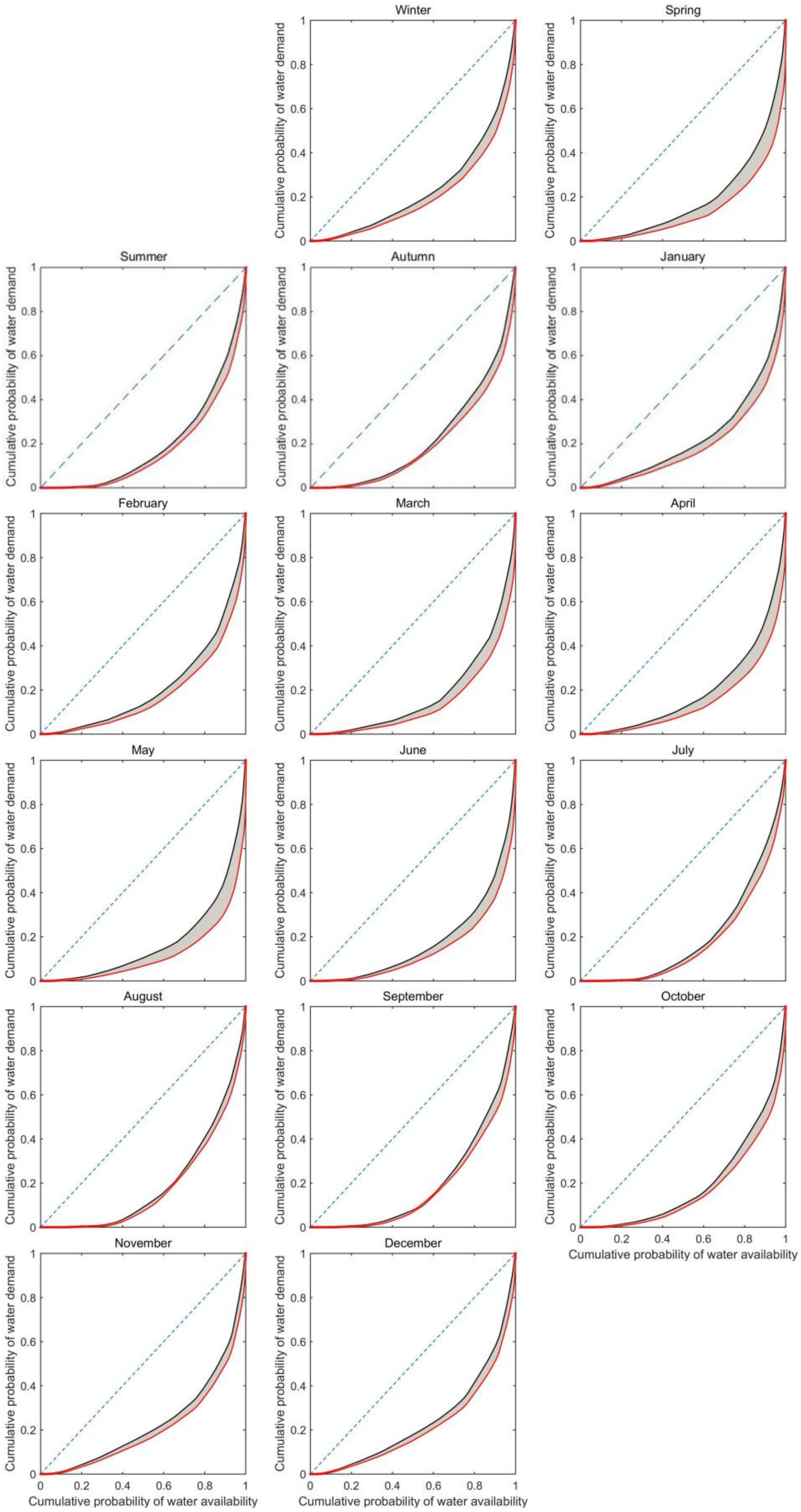

Supplementary Figure 8. Cumulative probability of water availability against cumulative portability of water withdrawals at the level of the second-order basin.

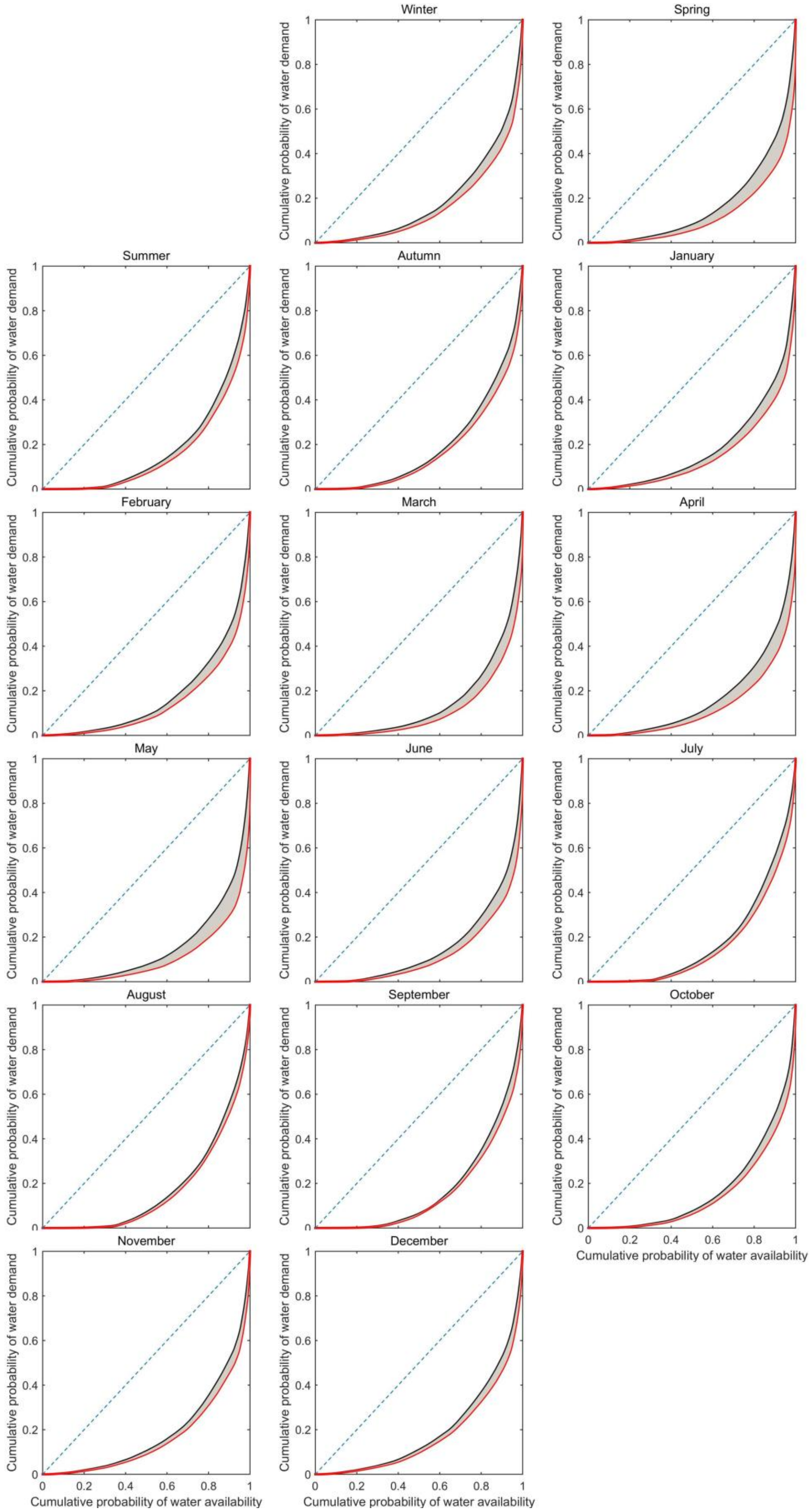

Supplementary Figure 9. Cumulative probability of water availability against cumulative portability of water withdrawals at the level of the third-order basin.

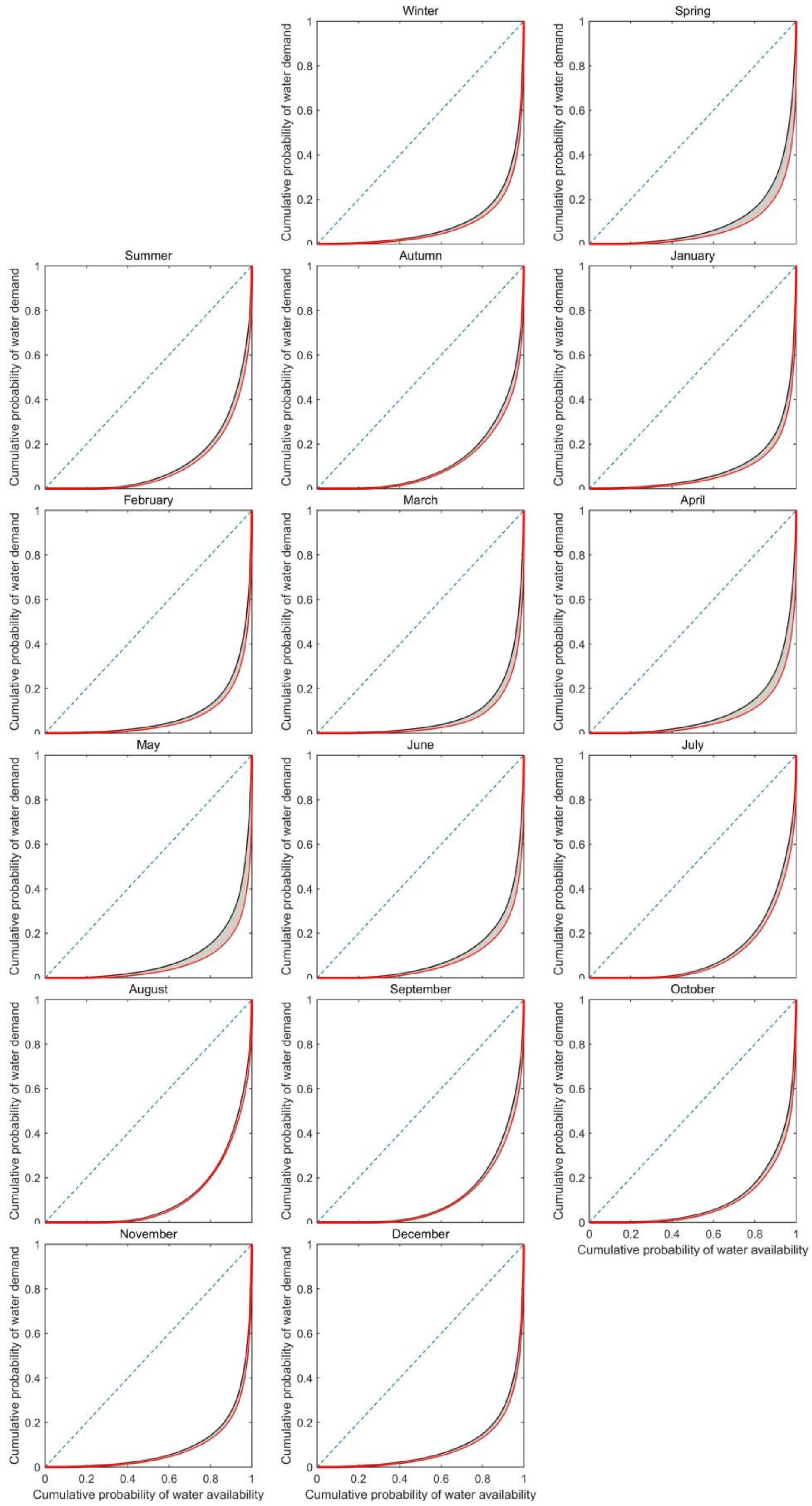

Supplementary Figure 10. Cumulative probability of water availability against cumulative portability of water withdrawals at the grid cell-level.

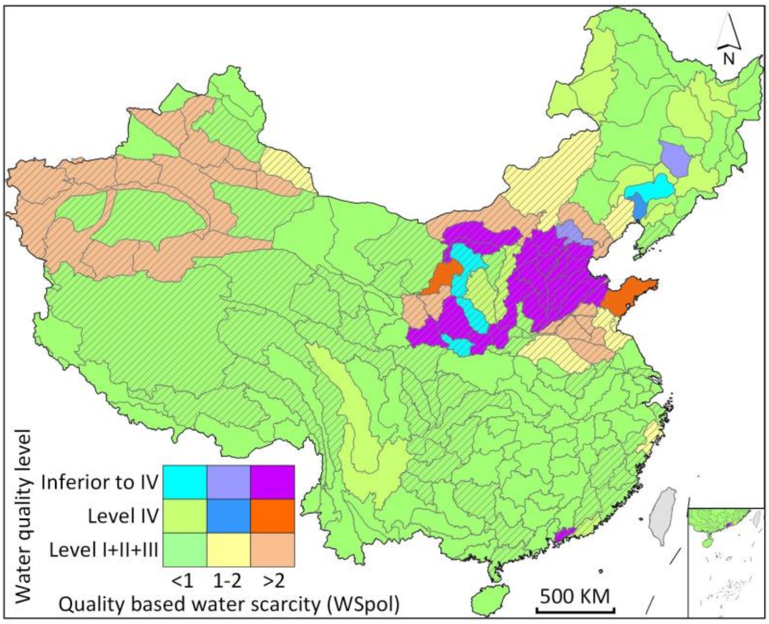

Supplementary Figure 11. Geographic distributions of surface water quality levels and quality based water scarcity (WSpol) across third-order basins in China. Regional water quality levels were determined by the annual average concentrations of the chemical oxygen demand and ammonium nitrogen during 2012-2016. Areas filled by solid lines indicate that where the annual average electrical conductivity is larger than 0.7 dS m^{-1}, the water quality threshold for the agricultural water use.

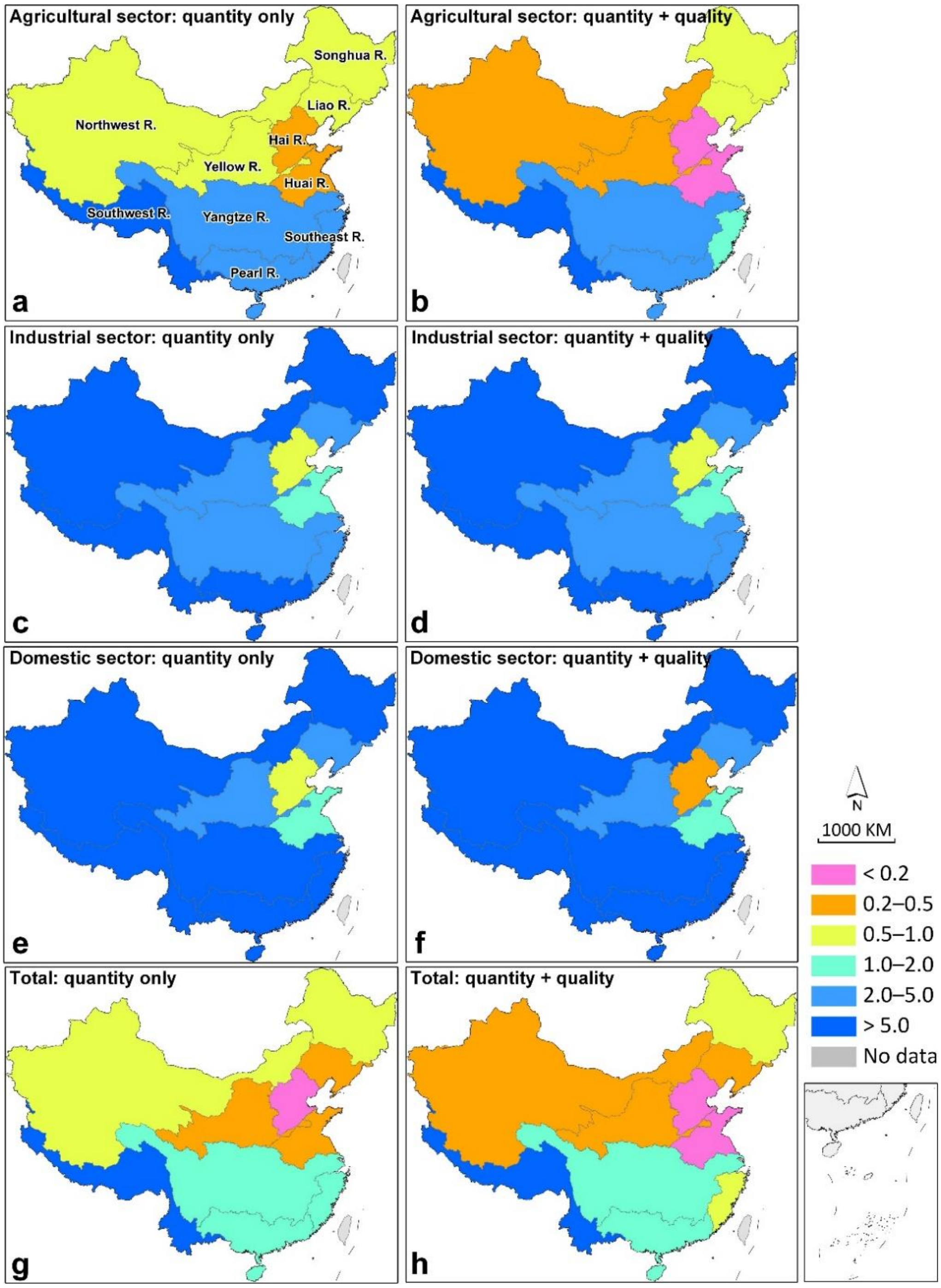

Supplementary Figure 12. Water carrying capacity in terms of a multiplier of present-day population and economic scales at the first-order basin level in China. The right panel (**b**, **d**, **f** and **h**) represents quantity based water carrying capacities for agricultural, industrial, domestic and all sectoral uses, respectively. The left panel (**a**, **c**, **e** and **g**) represents quality included water carrying capacities for agricultural, industrial, domestic and all sectoral uses, respectively. Source data are provided as a Source Data file.

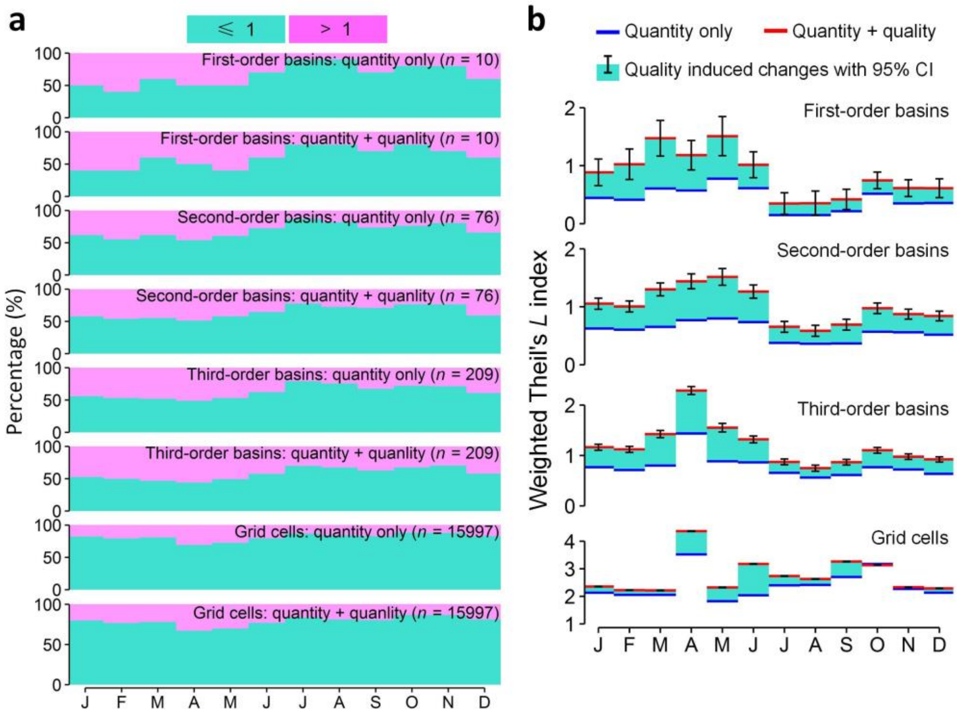

Supplementary Figure 13. Water scarcity on a monthly basis considering seasonal variability of environmental flow requirement. **a** The proportions of the number of basins under water scarcity. **b** Regional disparities in water scarcity indicated by Theil's *L* index. Source data are provided as a Source Data file.

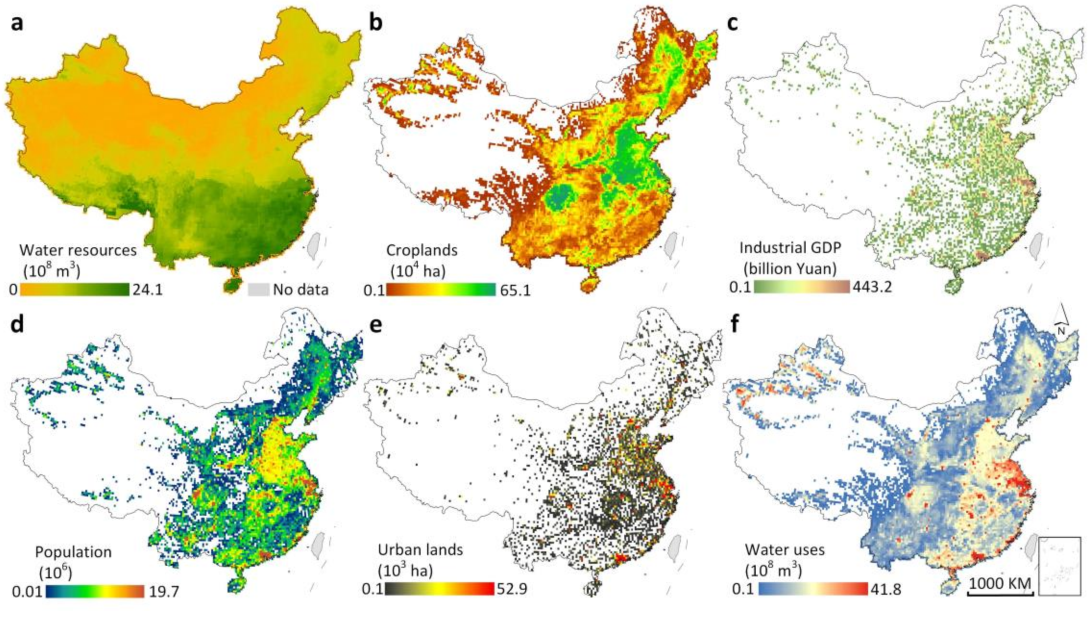

Supplementary Figure 14. Spatial distributions of variables used for water scarcity assessments at 0.25 × 0.25 arc-degree resolution. **a** Annual water resources availability. **b** Croplands including rice and non-rice croplands. **c** Industrial gross domestic product. **d** Population including rural and urban residents. **e** Urbanized lands. **f** Annual water withdrawal for irrigation, industry, domestic use and eco-environmental compensation demands. For all the variables, the mean of annual values for five years from 2012 to 2016 are presented.

Supplementary References

1. Adnane, Magri & Ewa Berezowska-Azzag. New tool for assessing urban water carrying capacity (WCC) in the planning of development programs in the region of Oran, Algeria. Sustain. *Cities Soc*. **10**, 13–16(2018).
2. Wang Y. et al. Evaluation of the comprehensive carrying capacity of interprovincial water resources in China and the spatial effect. *J. Hydrol* **575**, 794–809 (2019).
3. Liu, J. et al. Water scarcity assessments in the past, present and future. *Earth's Future* **5**, 545–559 (2017).
4. Pastor, A., Ludwig, F., Biemans, H., Hoff, H. & Kabat, P. Accounting for environmental flow requirements in global water assessments. *Hydrol. Earth Syst. Sci*. **18**, 5041–5059 (2014).
5. Liu, J. et al. Spatiotemporal characteristics, patterns, and causes of land-use changes in China since the late 1980s. *J. Geogr. Sci.* **24**, 195–210 (2014).
6. Jia, N. et al. Spatiotemporal patterns and characteristics of land-use change in China during 2010–2015. *J. Geogr. Sci.* **28**, 547–562 (2018).
7. Smith, M. CROPWAT: A computer program for irrigation planning and management. Food and Agriculture Organization of the United Nations (FAO), Rome (1992).
8. Döll, P. & Siebert, S. Global modeling of irrigation water requirements. *Water. Resour. Res.* **38**, 1037–1046 (2002).
9. Allen, R., Pereira, L., Raes, D. & Smith, M. Crop evapotranspiration: guidelines for computing crop requirements. FAO Irrigation and Drainage Paper No. 56. Food and Agriculture Organization of the United Nations (FAO), Rome (1998).
10. Peng, S., Ding, J., Mao, Z., Xu, J. & Li, D. Estimation and verification of crop coefficient for water saving irrigation of late rice using the FAO-56 method. *Transactions of the CSAE* **23**, 30–34 (2007). (In Chinese with English abstract).
11. Gao, Y., et al. Crop coefficient and water-use efficiency of winter wheat/spring maize strip intercropping. *Field Crop Res.* **111**, 65–73 (2009).
12. Liu, Y. & Luo, Y. A consolidated evaluation of the FAO-56 dual crop coefficient approach using the lysimeter data in the North China Plain. *Agr. Water Manage* **97**, 31–40 (2010).
13. Doll, C., Muller, J. & Elvidge, C. Night-time imagery as a tool for global mapping of socioeconomic parameters and greenhouse gas emissions. *Ambio* **29**, 157–162 (2000).
14. Chen, X. & Nordhaus, W. Using luminosity data as a proxy for economic statistics. *Proc. Natl. Acad. Sci. USA* **108**, 8589–8594 (2011).
15. Wada, Y. et al. Global monthly water stress: 2. Water demand and severity of water stress. *Water Resour. Res.* **47**, W07518 (2011).
16. Voisin, N. et al. One-way coupling of an integrated assessment model and a water resources model: evaluation and implications of future changes over the US Midwest. *Hydrol. Earth Syst. Sci.* **17**, 4555–4575 (2013).
17. Huang, Z. et al. Reconstruction of global gridded monthly sectoral water withdrawals for 1971–2010 and analysis of their spatiotemporal patterns. *Hydrol. Earth Syst. Sci.* **22**, 2117–2133 (2018).
