# Supplementary material for: Pollution exacerbates China’s water scarcity and its regional inequality
Source: Nat Commun. 2020 Jan 31;11:650. doi: 10.1038/s41467-020-14532-5 (PMC6994511; doi:10.1038/s41467-020-14532-5)
Supplement: Supplementary file 3 — Reporting Summary [file 41467_2020_14532_MOESM3_ESM.pdf]

natureresearch

Corresponding author(s): Ting Ma, Yong Ni, Chenghu Zhou

Last updated by author(s): Jan 17, 2020

Reporting Summary

Nature Research wishes to improve the reproducibility of the work that we publish. This form provides structure for consistency and transparency in reporting. For further information on Nature Research policies, see Authors & Referees and the Editorial Policy Checklist.

Statistics

For all statistical analyses, confirm that the following items are present in the figure legend, table legend, main text, or Methods section.

n/a	Confirmed	
☐	☒	The exact sample size (*n*) for each experimental group/condition, given as a discrete number and unit of measurement
☒	☐	A statement on whether measurements were taken from distinct samples or whether the same sample was measured repeatedly
☒	☐	The statistical test(s) used AND whether they are one- or two-sided<br>*Only common tests should be described solely by name; describe more complex techniques in the Methods section.*
☒	☐	A description of all covariates tested
☒	☐	A description of any assumptions or corrections, such as tests of normality and adjustment for multiple comparisons
☐	☒	A full description of the statistical parameters including central tendency (e.g. means) or other basic estimates (e.g. regression coefficient) AND variation (e.g. standard deviation) or associated estimates of uncertainty (e.g. confidence intervals)
☒	☐	For null hypothesis testing, the test statistic (e.g. *F*, *t*, *r*) with confidence intervals, effect sizes, degrees of freedom and *P* value noted<br>*Give P values as exact values whenever suitable.*
☒	☐	For Bayesian analysis, information on the choice of priors and Markov chain Monte Carlo settings
☒	☐	For hierarchical and complex designs, identification of the appropriate level for tests and full reporting of outcomes
☒	☐	Estimates of effect sizes (e.g. Cohen's *d*, Pearson's *r*), indicating how they were calculated

Our web collection on statistics for biologists contains articles on many of the points above.

Software and code

Policy information about availability of computer code

Data collection	No software was used.
Data analysis	R software (version 3.6.1). All computer codes used in the current study are available upon reasonable request.

For manuscripts utilizing custom algorithms or software that are central to the research but not yet described in published literature, software must be made available to editors/reviewers. We strongly encourage code deposition in a community repository (e.g. GitHub). See the Nature Research guidelines for submitting code & software for further information.

Data

Policy information about availability of data

All manuscripts must include a data availability statement. This statement should provide the following information, where applicable:

- Accession codes, unique identifiers, or web links for publicly available datasets
- A list of figures that have associated raw data
- A description of any restrictions on data availability

Detailed information regarding the data that support the findings of this study can be found in Supplementary Table 1. The source data underlying Figs. 1b-d, 2-4, and 5b and Supplementary Figs. 1-6, 12 and 13 are provided as a Source Datafile. The additional data that support the findings of this study are available from the corresponding author upon reasonable request.

October 2018

Field-specific reporting

Please select the one below that is the best fit for your research. If you are not sure, read the appropriate sections before making your selection.

☐ Life sciences ☐ Behavioural & social sciences ☒ Ecological, evolutionary & environmental sciences

For a reference copy of the document with all sections, see nature.com/documents/nr-reporting-summary-flat.pdf

Ecological, evolutionary & environmental sciences study design

All studies must disclose on these points even when the disclosure is negative.

Study description	In this study, we report a comprehensive nationwide water scarcity assessment in China, which explicitly includes quality requirements for human water uses cross four different geographic levels at three time-scales.
Research sample	We compiled nationwide datasets consisting of water availability, water quality and sector-specific water withdrawal for 15997 grid cells, 10 first-order basins, 76 second-order basins and 209 third-order basins across China.
Sampling strategy	No sampling.
Data collection	Three main types of data including water availability, water use and water quality were collected from China Water Resources Bulletin, China Statistical Yearbook, China Meteorological Data Network and the national environmental monitoring network, respectively. Ancillary data including land-use map and nighttime light data were provided by the Institute of Geographic Sciences and Natural Resources Research, the National Centers for Environmental Information (NCEI), respectively.
Timing and spatial scale	Timing scale: 2012-2016 on the annual, seasonal and monthly basis. Spatial scale: at the grid cell, the first-order, second-order and third-order basin scales.
Data exclusions	No data was excluded.
Reproducibility	The numerical results are fully reproducible from the description in the Methods section.
Randomization	Not relevant.
Blinding	Not relevant.

Did the study involve field work? ☐ Yes ☒ No

Reporting for specific materials, systems and methods

We require information from authors about some types of materials, experimental systems and methods used in many studies. Here, indicate whether each material, system or method listed is relevant to your study. If you are not sure if a list item applies to your research, read the appropriate section before selecting a response.

Materials & experimental systems

n/a	Involved in the study	
☒	☐	Antibodies
☒	☐	Eukaryotic cell lines
☒	☐	Palaeontology
☒	☐	Animals and other organisms
☒	☐	Human research participants
☒	☐	Clinical data

Methods

n/a	Involved in the study	
☒	☐	ChIP-seq
☒	☐	Flow cytometry
☒	☐	MRI-based neuroimaging
